# Solitary precaval right renal artery and bilateral double ureter in a patient with high-grade serous ovarian cancer

**DOI:** 10.1007/s00404-023-07090-w

**Published:** 2023-06-10

**Authors:** R. Schröder, M. K. Diener, F.-A. Taran

**Affiliations:** 1 Department of Obstetrics and Gynecology, University Medical Center Freiburg, Hugstetter Str. 55, 79106 Freiburg, Germany; 2 Department of General and Visceral Surgery, University Medical Center Freiburg, Freiburg, Germany

## Presentation

**Fig. 1:**
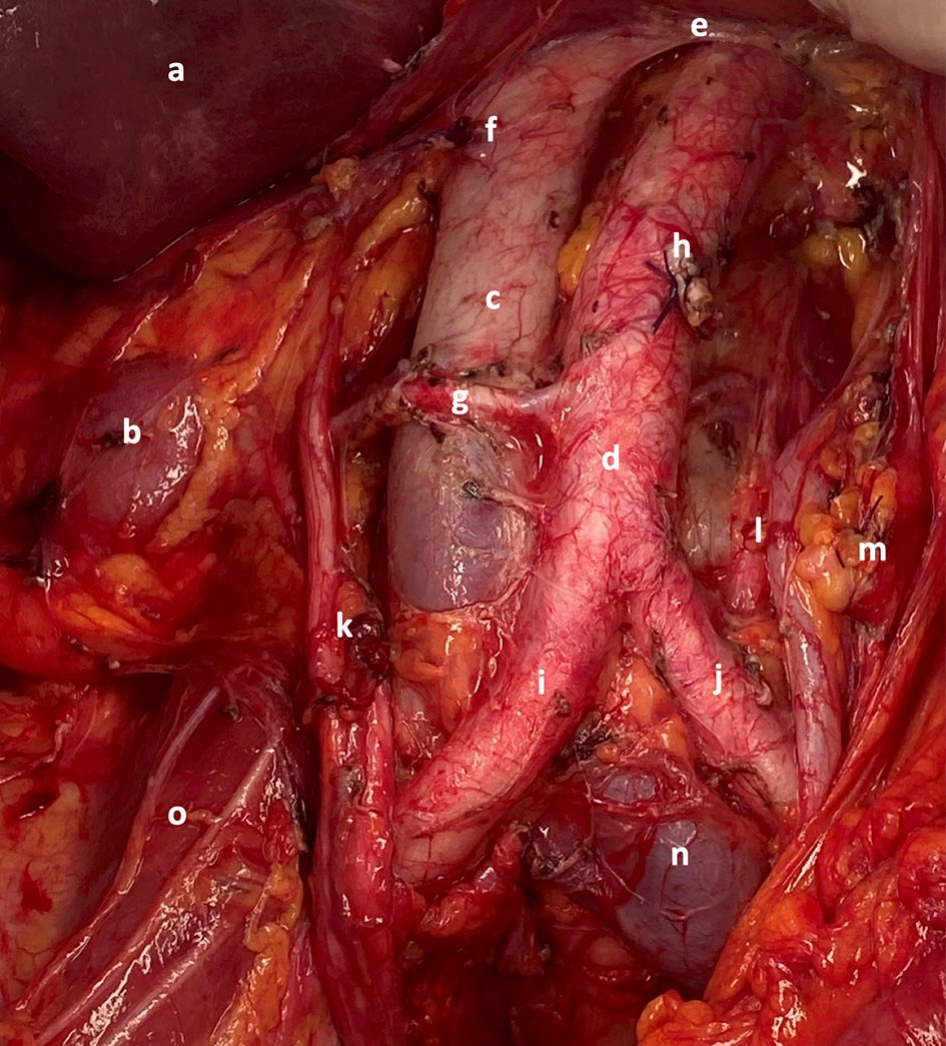
Solitary precaval right renal artery (RRA) originating from the anterior abdominal aorta (AA) below the inferior mesenteric artery (IMA), which has been dissected in order to achieve complete gross resection (**a** liver, **b** right kidney, **c** inferior vena cava, **d** abdominal aorta, **e** left renal vein, **f** right ovarian vein, **g** right renal artery, **h** inferior mesenteric artery, **i** right common iliac artery, **j** left common iliac artery, **k** right double ureter, **l** left double ureter, **m** left ovarian vein, **n** left common iliac vein, **o** right psoas muscle)

A 76-year old patient underwent primary cytoreductive surgery (CRS) for high-grade serous International Federation of Gynecology and Obstetrics (FIGO) stage IIIC ovarian cancer. Preoperative imaging by computed tomography described the presence of a solitary right renal artery (RRA), bilateral double ureter and pleviectasis of the right kidney. The CRS resulted in a and complete tumor resection and included: infragastric omentectomy, radical hysterectomy, bilateral salpingo-oophorectomy, partial peritonectomy (pelvis, upper abdomen and left diaphragmatic region), para-aortic lymphadenectomy for bulky lymph nodes (up to 4 cm in diameter), right hemicolectomy, anterior resection of the rectosigmoid, cholecystectomy, splenectomy and full-thickness resection of the right diaphragm. Preparation of the abdominal aorta up until the origination of the left renal vein showed a solitary precaval RRA and a bilateral double ureter (Fig. 1). The patient recovered well after the procedure.

## Discussion

The RRA passes usually behind its accompanying vein and the inferior vena cava [1]. However, there a large number of anatomical variants of the renal artery (RA), with an additional RA being the most common one [1]. A precaval RRA has a reported prevalence of up to 5% [2, 3]. The majority of precaval RRA appear to be additional arteries with only 10 published cases of solitary precaval RRA [3]. An association with pelvieactasis as described in our patient has also been reported previously [2]. Additionally, a strong relation between a lower origin of the RRA and a precaval course has been described [4]. A correlation between a double ureter and precaval RRA is linked to an embryologic origin [3, 5]. Complex oncological surgery including multivisceral resections and extended multi-field lymphadenectomy requires in-depth anatomical knowledge, including the presented anatomical varieties of the RRA. As in this case shown, pre-operative imaging should be used in order to identify and/or explicitly mention anatomical variations.
